# Dynamic Echocardiographic Changes Induced by Exercise in Healthy, Young Individuals with Early Repolarization Pattern

**DOI:** 10.3390/diagnostics15141755

**Published:** 2025-07-11

**Authors:** Loránd Kocsis, Zsuzsanna Pap, István Adorján Szabó, Attila Frigy

**Affiliations:** 1 Doctoral School of Medicine and Pharmacy, George Emil Palade University of Medicine, Pharmacy, Science and Technology of Targu Mures, 540142 Targu Mures, Romania; 2 Department of Anatomy and Embryology, George Emil Palade University of Medicine, Pharmacy, Science and Technology of Targu Mures, 540142 Targu Mures, Romania; 3 Department of Cardiology, Dr. Szabo Medical Center, 545200 Ludus, Romania; 4 Department of Internal Medicine IV, George Emil Palade University of Medicine, Pharmacy, Science and Technology of Targu Mures, 540103 Targu Mures, Romania; 5 Department of Cardiology, Clinical County Hospital Mures, 540103 Targu Mures, Romania

**Keywords:** early repolarization, exercise test, echocardiography, arrhythmia

## Abstract

**Background**: The early repolarization pattern (ERP) on electrocardiography (ECG) has been associated with an increased risk of ventricular arrhythmias in susceptible individuals. This study aimed to evaluate the impact of exercise on echocardiographic parameters to explore the potential influence of ERP on hemodynamic response. **Methods**: Twenty-five healthy, young males with ERP (ERP+ group) and 25 age-matched healthy males without ERP (ERP− group) were enrolled. Comprehensive transthoracic echocardiography was performed at rest and during the early recovery phase following a treadmill exercise test. Baseline values and exercise-induced changes in both conventional and strain-derived echocardiographic parameters were analyzed and compared between groups. **Results**: Anthropometric measures and resting vital signs were similar in both groups. At baseline, the ERP+ group had a shorter QRS duration. Both groups demonstrated excellent cardiovascular fitness, with comparable chronotropic and pressor responses to exercise. Resting and early recovery-phase echocardiographic parameters were largely similar between ERP+ and ERP− individuals, with no overt structural or functional abnormalities observed in either group. However, ERP+ individuals showed significantly greater reductions in left ventricular end-diastolic volume and stroke volume following exercise, suggesting a distinct volumetric response to physical stress. **Conclusions**: ERP in healthy young males is not associated with structural cardiac abnormalities or overt myocardial dysfunction. The observed exercise-induced volumetric changes may indicate subtle differences in hemodynamic adaptation, warranting further investigation.

## 1. Introduction

**Figure 1:**
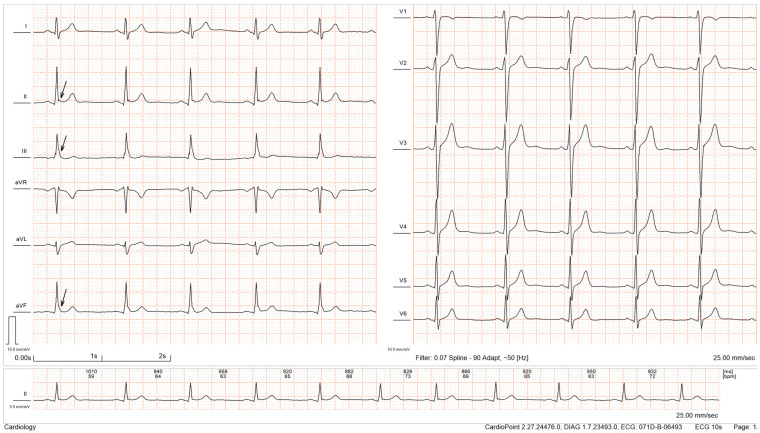
ECG recording with ERP in the inferior leads, showing prominent J waves (marked by arrows) (from the authors’ personal collection).

The early repolarization pattern (ERP) consists of an abnormality seen on the electrocardiogram (ECG), at the end of the QRS complex, known as the J wave (Figure 1). Although it was considered a benign ECG phenomenon, recent studies have shown that ERP can be associated with life-threatening cardiac arrhythmias (early repolarization syndrome—ERS) and sudden cardiac death (SCD) [1]. The 2022 ESC Guidelines for the management of ventricular arrhythmias and the prevention of sudden cardiac death define ERP as a distinct ECG pattern and recommend considering its presence in the risk assessment of patients with idiopathic ventricular fibrillation [2].

The prevalence of ERP is relatively high in young males, reaching up to 31%, and occurs in 70% of individuals who have experienced idiopathic ventricular fibrillation [3,4].

The precise mechanism behind the formation of the J wave is not fully understood. Antzelevitch et al. [1] propose a mechanism involving a larger notch in the initial phase of the action potential occurring in the epicardium compared to the endocardium. This is partly due to an increased transient outward potassium current, caused by genetic mutation, leading to a higher transmural voltage gradient that appears as a J wave on the surface ECG. Additionally, abnormalities in sodium, calcium, and ATP-dependent potassium channels can also result in J waves. According to Haissaguerre et al. [5], structural abnormalities may also contribute to the formation of the J wave by delaying impulse conduction in the epicardium (“late depolarization” theory). In line with these mechanisms, Badura et al. recently classified ERS among the primary electrical heart diseases (channelopathies), highlighting its potential genetic and electrophysiological overlap with other syndromes, such as Brugada or short QT [6].

A recent study by Morita et al. identified specific ECG features—such as QRS widening, fragmented QRS, and wide J waves—as predictors of ventricular fibrillation in patients with ERS, suggesting that certain ECG patterns may reflect underlying conduction or structural substrates [7]. Based on ECG and electrophysiological data, the inferior and/or lateral regions of the left ventricle are involved in the genesis of ERP [8]. ERS shares several clinical similarities with the Brugada syndrome (BrS), where the specific J-wave pattern appears in the right precordial leads on the surface ECG. According to the latest consensus report, these two entities are considered the two forms of the J-wave syndromes [9]. Studies on BrS patients have identified structural changes in the right ventricular outflow tract, such as increased collagen content and fibrosis of the myocardium, abnormal myocardial expression of connexin-43, keratin-24, α-cardiac actin, or α-skeletal actin [10]. Additionally, imaging studies in BrS have identified structural and functional abnormalities, such as right ventricular dilation, abnormal right ventricular ejection fraction, delayed contraction of the right ventricular outflow tract, pathological strain values, and tissue characteristics. These structural changes are more commonly found in patients who have experienced SCD [11,12]. These findings raise the question of whether ERP in otherwise healthy individuals might also be associated with subtle morphological or functional cardiac changes.

We hypothesized that individuals with ERP may exhibit morphological and functional cardiac changes, similar to those described in BrS, particularly in the myocardial regions involved in its pathomechanism. In this study, we used a detailed echocardiographic evaluation both at rest and during the early recovery phase after exercise to identify potential morphological and functional responses, with possible clinical significance in individuals with ERP. To our knowledge, this is the first study to assess echocardiographic changes in ERP-positive individuals in response to exercise, which represents the novelty of this investigation. The aim of this study was to evaluate morphological and functional echocardiographic parameters before and after exercise in ERP-positive individuals, in order to explore whether ERP influences the heart’s functional or hemodynamic response.

## 2. Materials and Methods

### 2.1. Study Population

In a prospective study, we included 25 young, healthy males (mean age 22.9 ± 1.7 years) diagnosed with ERP (ERP+ group) based on the criteria published by Macfarlane et al. [13] Additionally, a control group of 25 individuals (mean age 22.2 ± 1.7 years) without ERP (ERP− group) was included. Each participant provided signed informed consent for participation and underwent a detailed medical history and physical examination. All participants were free of known diseases, including cardiovascular conditions, had normal physical examination results, and were not on any active medications.

This study was approved by the Ethical Committee of Research at George Emil Palade University of Medicine, Pharmacy, Science, and Technology of Targu Mures, Romania (CEC 129/2018).

### 2.2. Echocardiographic Imaging and Analysis

Each participant underwent a detailed echocardiographic examination. A resting echocardiographic examination was conducted at first in the standard left lateral decubitus position, followed by an exercise test, with a second echocardiographic examination performed during the early recovery phase (within approximately 1–2 min after the cessation of exercise), also in the left lateral decubitus position. We used a Philips Epiq7 device (Philips Healthcare, Amsterdam, The Netherlands) with a S5-1 sector array transducer. During the examination, parasternal long-axis and short-axis views, and apical 4-chamber, 2-chamber, and 3-chamber views were saved, as well as parameters characterizing the diastolic function of the left ventricle. Echocardiographic data were recorded by the same expert cardiologist. The data were saved and processed offline using the QLAB Cardiac Analysis software package (Philips Healthcare, Amsterdam, The Netherlands). The measurements of heart cavity diameters, as well as systolic and diastolic parameters, were performed in accordance with the current guidelines [14].

In brief, measurements of left ventricular dimensions (LVEDD—left ventricular end-diastolic diameter, LVESD—left ventricular end-systolic diameter), wall thicknesses (IVST—interventricular septum thickness at end-diastole, PWT—left ventricular posterior wall thickness at end-diastole), left atrium antero-posterior diameter (LAD), and the aortic root (AoR) were obtained in the parasternal long-axis view. The diastolic function (E—peak early-diastolic transmitral flow velocity, A—peak late-diastolic transmitral flow velocity), and right ventricle basal diameter (RVD—right ventricle diameter, apical 4-chamber) were assessed in the apical four-chamber view.

The left ventricular volumes (LVEDV—end-diastolic volume, LVESV—end-systolic volume), ejection fraction (EF), and stroke volume (SV) were calculated using the methods implemented in the QLab Cardiac Analysis software package, utilizing automated two-dimensional cardiac quantification (a2DQ). Additionally, strain measurements were performed, GLS (global longitudinal strain), using automated cardiac motion quantification (aCMQ) based on 2D speckle tracking analysis.

Diastolic function was measured using pulsed-wave Doppler across the mitral valve leaflets. Left ventricular mass was calculated as 0.8 × [1.04 × ((LVEDD + IVST + PWT)^3^ − (LVEDD)^3^)] + 0.6, and relative wall thickness was determined as (IVST + PWT)/LVEDD. The shortening fraction (SF) was calculated using the following formula: SF (%) = ((LVEDD − LVESD)/LVEDD) × 100.

### 2.3. Treadmill Exercise Testing

Participants were instructed to abstain from alcohol, coffee, and any stimulating nutritional supplements for at least 6 h before the exercise test. For each participant, we recorded a resting standard 12-lead ECG. Following this, they underwent a treadmill exercise test using the Bruce protocol, continuing until they reached at least 85% of their age-predicted maximum heart rate. The protocol began at a speed of 2.7 km/h and a 10% incline in Stage 1, with each stage increasing in intensity, progressing up to 9.6 km/h and a 22% incline by Stage 7, corresponding to increasing levels of exertion (up to 23.7 METs). Throughout the test, continuous ECG monitoring and intermittent non-invasive blood pressure measurements were performed under the supervision of a cardiologist.

The ECG data were collected using a BTL-08 SD3 ECG device, connected to a desktop computer running BTL CardioPoint software, version 2.27.24476.0. The device featured a sampling frequency of 2000 Hz, a digital resolution of 3.9 μV, and a 13 bit A/D conversion.

### 2.4. Statistical Analysis

Descriptive and interferential statistical analyses were conducted. Data are presented as mean ± standard deviation (continuous variables) or as percentages (categorical variables). Normality was assessed using the Shapiro–Wilk test. Between-group comparisons (ERP+ vs. ERP−) were performed using independent samples T-test or Mann–Whitney U test, as appropriate. Categorical variables were expressed as percentages, and differences between groups were evaluated using the Chi-square test or Fisher’s exact test as appropriate. A *p*-value of less than 0.05 was considered statistically significant.

Data analysis was performed using Microsoft Excel, version 2402 (Microsoft Corporation, Redmond, WA, USA), and IBM SPSS Statistics, version 25 (IBM, Armonk, NY, USA).

## 3. Results

### 3.1. Clinical Characteristics

In the comparative analysis of the clinical characteristics between the ERP+ and ERP− groups, no significant differences were found in age or anthropometric measures, such as weight, height, body surface area, and body mass index (Table 1).

During the evaluation of vital signs, the mean resting systolic and diastolic blood pressure levels were close to the optimal range for both groups, with no significant differences detected. Heart rates were within the normal range in both groups, and no significant differences were observed between the groups (Table 1).

### 3.2. ECG Characteristics

Table 2 summarizes the baseline ECG parameters of the ERP+ and ERP− groups. The P wave duration was longer in the ERP+ group, but the difference was not significant. The P wave amplitude was within the normal range, with no significant difference between the groups. The PQ interval was similar in both groups and remained within the normal range. The QRS axis was intermediate with similar values in the groups. All participants had a QRS duration of less than 120 ms, with the ERP+ group showing a significantly shorter QRS duration (91 ± 7 ms vs. 97 ± 7 ms, *p* = 0.017). No supraventricular or ventricular premature beats were observed on the registered ECGs. The ST segment was isoelectric in all individuals, and the QTc interval was identical in both groups, with values within the normal range. These findings are in line with the results of a more detailed comparative analysis of ECG parameters in ERP+ vs. ERP- individuals performed by our study group [15].

### 3.3. Treadmill Exercise Test Results

Regarding the exercise-induced physiological responses (Table 3), both ERP+ and ERP− groups demonstrated high MET values. All participants, except for two in the ERP+ group and one in the ERP− group, surpassed the 12 MET threshold, indicating excellent exercise capacity. The average peak heart rates were 188 bpm for the ERP+ group and 187 bpm for the ERP− group. The pressor response, reaching a peak systolic blood pressure of 160 mmHg and a modest increase in diastolic blood pressure, was within normal limits for this population.

### 3.4. Echocardiographic Characteristics

The resting echocardiographic assessment revealed no pathological findings in either group. As shown in Table 4, baseline morphological and functional parameters—including left ventricular dimensions (LVEDD, LVESD), wall thickness (IVST, PWT), left ventricular mass (LVM), and volumes (LVEDV, LVESV)—were comparable between ERP+ and ERP− individuals. Indices of systolic function, such as EF, SF, SV, and GLS, as well as parameters of diastolic function (E, A, and E/A), showed no significant differences. Similarly, other morphological parameters, including LAD, AoR, and RVD, were also comparable between the groups.

Early recovery-phase echocardiographic measurements (Table 5) revealed no statistically significant differences between ERP+ and ERP− groups across the assessed parameters. While baseline values of all parameters reflecting left ventricular filling and systolic function were similar between groups, a post-exercise trend toward lower values was observed in the ERP+ group for LVEDV, LVESV, and SV: LVEDV was 107.7 ± 18.8 mL vs. 118.3 ± 19.1 mL (*p* = 0.058), LVESV was 38.9 ± 7.3 mL vs. 43.3 ± 7.4 mL (*p* = 0.067), and SV was 67.5 ± 14.1 mL vs. 75.0 ± 14.4 mL (*p* = 0.053). All other echocardiographic parameters—including wall thicknesses, GLS value, EF, and diastolic function—remained comparable between groups. The data regarding the resting echocardiographic parameters correspond to the more detailed analysis performed by our team in ERP+ vs. ERP− individuals [16].

**Figure 2:**
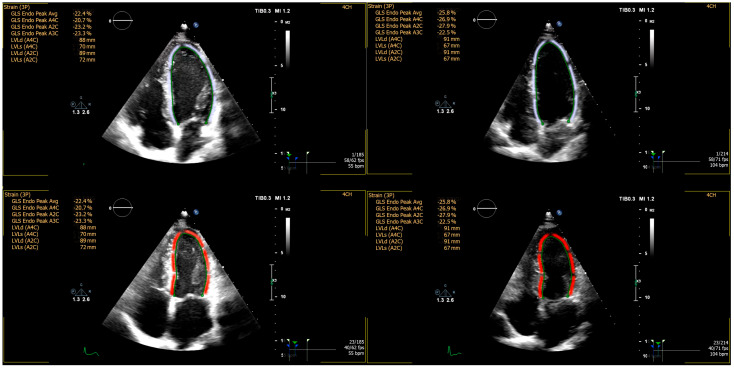
Global longitudinal strain (GLS) assessed by speckle-tracking echocardiography. The figure shows GLS measurements for a healthy, young individual with ERP, demonstrating a GLS of −22.4% at a heart rate of 55 bpm (**left side**) and −25.8% at a heart rate of 104 bpm (**right side**). The images display regional strain maps from two-dimensional echocardiography in apical four-chamber views. The upper images represent the heart at end-diastole, while the lower images show it at end-systole. No regional or global wall motion abnormalities are observed.

The exercise-induced changes in echocardiographic parameters (Δ values) are presented in Table 6. A consistent trend, similar to that observed in certain early recovery-phase parameters, was evident in the ERP+ group, with significantly greater reductions in LVEDV (−10.1 ± 11.8 mL vs. −1.1 ± 13.2 mL, *p* = 0.020) and SV (−4.8 ± 9.9 mL vs. 1.4 ± 8.9 mL, *p* = 0.031) compared to the ERP− group. Additionally, the reduction in LVESV (−6.6 ± 5.4 mL vs. −3.0 ± 5.5 mL, *p* = 0.075) approached statistical significance. Changes in other echocardiographic parameters, including indices of systolic function (Δ EF, Δ SF), myocardial strain (Δ GLS, Figure 2), and diastolic function (Δ E, Δ A, Δ E/A), were comparable between groups. Similarly, structural adaptations of the left ventricle (Δ LVEDD, Δ LVESD, Δ IVST, Δ PWT) and other cardiac dimensions (Δ LAD, Δ AoR, Δ RVD) did not differ significantly between ERP+ and ERP− individuals.

## 4. Discussion

Our findings contribute to the current knowledge related to the study of cardiac characteristics in individuals with ERP. Previous studies, both on ERP/ERS and on Brugada syndrome, have suggested that the presence of J wave may be associated with morphological and functional myocardial abnormalities [17,18,19].

Exercise testing revealed excellent exercise tolerance in both groups, surpassing the 12 MET threshold. No significant differences were observed between ERP+ and ERP− individuals in terms of exercise duration, heart rate response, or blood pressure dynamics. The cardiovascular responses remained within the expected physiological range for high-intensity exertion in the young adult males [20]. The presence of ERP did not influence chronotropic or pressor responses during physical activity and did not affect exercise performance.

A detailed echocardiographic assessment was performed for demonstrating the appearance of cardiac alterations induced by submaximal exercise in ERP-positive individuals—an area that is still largely unexplored in the current literature.

Morphological echocardiographic parameters showed no significant differences between ERP+ and ERP− groups in terms of left ventricular dimensions, wall thicknesses, ventricular mass, or other structural parameters characterizing the left atrium, right ventricle, or aortic root. This similarity persisted in measurements taken during the early recovery phase, and the exercise-induced changes in these parameters were also comparable between the two groups. Previous studies have yielded varied results regarding ventricular morphology and function in individuals with ERP. Among the larger-scale investigations, the study by Trenkwalder et al. is notable for reporting of decreased LVEDD, LVESD, and LVM in ERP-positive males based on echocardiographic measurements [21]. In contrast, several studies involving athletic populations have reported opposing findings, with ERP+ individuals showing an increased LVM [22,23] and LVEDD [22,24], while LVESD values remained similar between ERP+ and ERP− groups [23,25]. Notably, the Dallas Heart Study, which utilized MRI assessments in a large-scale population, also report a significantly greater LVM in ERP-positive subjects [26]. Some other smaller-scale studies have not identified significant differences in most echocardiographic morphological parameters between ERP+ and ERP− individuals [27,28].

**Figure 3:**
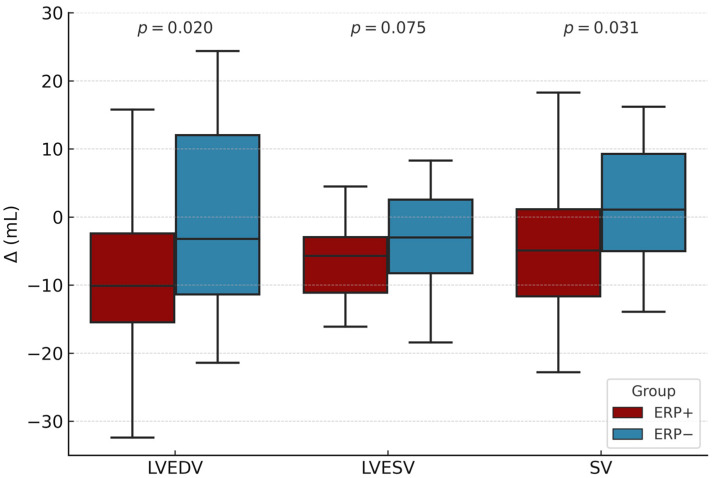
Boxplot showing exercise-induced changes in LVEDV, LVESV, and SV for ERP+ (dark red) and ERP− (blue) groups. The *y*-axis represents absolute changes (Δ) in milliliters. Boxes indicate the interquartile range (IQR), with the median marked by a horizontal line.

Regarding left ventricular systolic and diastolic function, similar characteristics were observed in our population both at baseline and during the early recovery phase. Although, early recovery-phase values of LVEDV, LVESV, and SV did not differ significantly between ERP+ and ERP− individuals, a consistent trend toward lower values was noted in the ERP+ group. This may reflect a more pronounced reduction in ventricular filling, residual systolic volume, and stroke output in response to exercise. Notably, both LVEDV and SV exhibited statistically significant differences in their exercise-induced changes (Δ values, Figure 3), suggesting a potentially blunted hemodynamic response in ERP+ individuals under physical stress. These findings are in line with the results of Trenkwalder et al., who reported lower LVEDV, LVESV, and SV in ERP-positive subjects [21]. In contrast, a relative smaller-scale study by Miragoli et al. found comparable volume-related indices between ERP+ and ERP− individuals [23]. Other markers of systolic function (EF and SF) and diastolic performance (E, A, and E/A) remained similar across groups at baseline and after exercise, reinforcing the absence of overt myocardial dysfunction in ERP-positive individuals. These results are consistent with previous papers describing preserved systolic and diastolic myocardial function in the ERP population [22,25].

GLS, assessed by speckle-tracking echocardiography, is a sensitive marker of myocardial systolic performance, and is particularly able to detect early abnormalities in ventricular longitudinal function. In our study, GLS values were comparable between ERP+ and ERP− individuals, both at baseline and the early recovery phase, with no exercise-induced alterations suggestive of myocardial dysfunction. These results are consistent with the findings of Gulel et al., who reported preserved global deformation in ERP-positive subjects [28]. In contrast, Colluoglu et al. described lower GLS values in ERP individuals, especially in the inferolateral and basal segments, suggesting that ERP may, in some cases, be associated with subtle regional impairment of longitudinal function. Their findings further propose that ERP could reflect not only ion channel abnormalities but also mechanical dysfunction, potentially contributing to arrhythmogenic risk [29].

Our study has several limitations that should be acknowledged. Firstly, the relatively small sample size may limit statistical power and generalizability. Secondly, our exclusive inclusion of young, healthy males restricts the applicability of results to broader populations, including females, older individuals, and patients with underlying cardiovascular diseases. Additionally, although echocardiography provides valuable insights into cardiac morphology and function, more sensitive and advanced imaging techniques, such as cardiac magnetic resonance imaging, might detect more subtle structural changes. Moreover, certain additional echocardiographic parameters, such as E/e′, deceleration time, and TAPSE, were not included in our protocol, which may limit the assessment of diastolic and right ventricular function. Addressing these limitations in future studies will be essential for achieving a comprehensive understanding of ERP’s clinical and cardiac morpho-functional implications.

## 5. Conclusions

We performed a comprehensive echocardiographic evaluation of healthy young individuals with ERP, both at rest and during the early recovery phase. Our findings reinforce that ERP is not associated with pathological cardiac morphology or overt systolic or diastolic dysfunction. While most echocardiographic parameters remained comparable between ERP+ and ERP− groups, exercise-induced changes revealed significantly greater reductions in LVEDV and SV among ERP+ individuals, with a similar trend observed for LVESV. These results may reflect subtle differences in cardiac hemodynamic adaptation to physical stress in ERP-positive individuals. The preserved global systolic function and GLS suggest that ERP does not associate significant myocardial dysfunction in this population. Further large-scale and longitudinal studies are warranted to clarify the potential clinical implications of the functional pattern evidenced by our study.

## Figures and Tables

**Table 1:** Physiological measurements at the physical examination before the exercise test.

	ERP+	ERP−	*p*
**Anthropometric measurements**			
Age, years	22.9 ± 1.7	22.2 ± 1.7	0.171
Weight, kg	76.9 ± 9.7	74.4 ± 9.2	0.393
Height, cm	179 ± 6	178 ± 6	0.579
Body surface area, m^2^	1.95 ± 0.14	1.92 ± 0.14	0.385
Body mass index, kg/m^2^.	23.9 ± 2.5	23.3 ± 2.3	0.449
**Vital signs**			
Systolic blood pressure, mmHg	123 ± 13	123 ± 13	0.941
Diastolic blood pressure, mmHg	79 ± 7	78 ± 7	0.777
Heart rate, bpm	68 ± 9	72 ± 9	0.227

Data are presented as mean ± standard deviation.

**Table 2:** The baseline ECG parameters of the individuals with and without ERP.

	ERP+	ERP−	*p*
RR interval, ms	902 ± 140	864 ± 140	0.410
P wave duration, ms	108 ± 10	102 ± 10	0.0810
PQ(PR) interval, ms	158 ± 16	166 ± 16	0.215
P wave amplitude, mm	1.2 ± 0.4	1.3 ± 0.4	0.527
QRS axis, grade	60 ± 24	64 ± 24	0.578
QRS duration, ms	91 ± 7	97 ± 7	0.017
QT interval corrected, ms	385 ± 26	381 ± 27	0.543
Significant ST segment modification, %	0	0	-
Atrial premature beats, %	0	0	-
Ventricular premature beats, %	0	0	-

Data are presented as mean ± standard deviation (continuous variables) or as percentages (categorical variables). The corrected QT intervals were calculated using Bazett’s formula.

**Table 3:** Treadmill exercise testing parameters.

	ERP+	ERP−	*p*
METs	14.8 ± 2.6	16.9 ± 2.6	0.110
Duration of exercise, min	11.6 ± 2.4	12.4 ± 2.4	0.465
Heart rate at peak, bpm	188 ± 8	187 ± 8	0.620
Systolic blood pressure at peak, mmHg	164 ± 18	160 ± 18	0.515
Diastolic blood pressure at peak, mmHg	90 ± 9	86 ± 9	0.137

Data are presented as mean ± standard deviation. The MET values correspond to the level of exercise that was necessary to reach the target heart rate.

**Table 4:** Resting parameters obtained during echocardiography before the exercise test.

	ERP+	ERP−	*p*
**Left ventricular morphological parameters**			
LVEDD, mm	45.9 ± 3.7	46.8 ± 3.7	0.287
LVESD, mm	30.7 ± 3.8	31.7 ± 3.9	0.333
IVST, mm	9.3 ± 0.7	9.1 ± 0.7	0.376
PWT, mm	9.3 ± 0.6	9.4 ± 0.6	0.724
RWT, %	41.0 ± 4.3	40.2 ± 4.4	0.505
LVEDV, mL	118.1 ± 15.9	119.4 ± 14.3	0.783
LVESV, mL	45.4 ± 7.8	46.2 ± 7.3	0.728
LVM, g	144.7 ± 24.5	148.3 ± 24.6	0.601
**Left ventricular systolic and diastolic parameters**			
EF, %	61.5 ± 3.3	61.3 ± 3.3	0.853
SF, %	33.1 ± 5.9	32.1 ± 6.0	0.633
GLS	−23.0 ± 1.3	−22.5 ± 1.3	0.177
SV, mL	72.4 ± 9.5	73.5 ± 9.5	0.691
E, cm/s	87.2 ± 16.4	88.0 ± 16.7	0.876
A, cm/s	52.2 ± 10.6	50.6 ± 10.9	0.616
E/A	1.7 ± 0.4	1.8 ± 0.4	0.815
**Other morphological parameters**			
LAD, mm	31.4 ± 4.9	30.7 ± 4.8	0.590
AoR, mm	21.2 ± 1.3	21.7 ± 1.4	0.187
RVD, mm	36.0 ± 3.7	34.8 ± 3.7	0.277

Data are presented as means ± standard deviation. LVEDD—left ventricular end-diastolic diameter; LVESD—left ventricular end-systolic diameter; IVST—interventricular septum thickness at end-diastole; PWT—left ventricular posterior wall thickness at end-diastole; RWT—relative wall thickness; LVM—left ventricular mass; LVEDV—left ventricular end-diastolic volume; LVESV—left ventricular end-systolic volume; EF—ejection fraction; SF—shortening fraction; GLS—global longitudinal strain; SV—stroke volume; E—peak early-diastolic transmitral flow velocity; A—peak late-diastolic transmitral flow velocity; LAD—left atrial diameter (antero-posterior); AoR—aortic root dimension; RVD—right ventricular dimension (basal).

**Table 5:** Echocardiographic morphological and functional parameters obtained during the early recovery phase.

	ERP+	ERP−	*p*
**Left ventricular morphological parameters**			
LVEDD, mm	46.2 ± 3.9	47.2 ± 3.9	0.352
LVESD, mm	26.1 ± 3.8	27.1 ± 3.8	0.322
IVST, mm	9.1 ± 0.8	9.0 ± 0.8	0.625
PWT, mm	9.2 ± 0.9	9.1 ± 1.0	0.876
RWT, %	39.9 ± 5.4	38.8 ± 5.5	0.726
LVEDV, mL	107.7 ± 18.8	118.3 ± 19.1	0.058
LVESV, mL	38.9 ± 7.3	43.3 ± 7.4	0.067
**Left ventricular systolic and diastolic parameters**			
EF, %	64.0 ± 3.0	63.4 ± 3.0	0.500
SF, %	43.6 ± 6.1	42.3 ± 6.2	0.503
GLS	−24.8 ± 1.7	−22.3 ± 1.7	0.262
SV, mL	67.5 ± 14.1	75.0 ± 14.4	0.053
E, cm/s	106.5 ± 15.7	110.2 ± 15.4	0.440
A, cm/s	69.2 ± 17.2	75.4 ± 17.2	0.201
E/A	1.6 ± 0.3	1.5 ± 0.3	0.212
**Other morphological parameters**			
LAD, mm	31.9 ± 3.9	30.1 ± 3.7	0.106
AoR, mm	21.2 ± 1.3	21.7 ± 1.3	0.233
RVD, mm	35.9 ± 4.3	34.6 ± 4.2	0.372

Data are presented as means ± standard deviation. LVEDD—left ventricular end-diastolic diameter; LVESD—left ventricular end-systolic diameter; IVST—interventricular septum thickness at end-diastole; PWT—left ventricular posterior wall thickness at end-diastole; RWT—relative wall thickness; LVEDV—left ventricular end-diastolic volume; LVESV—left ventricular end-systolic volume; EF—ejection fraction; SF—shortening fraction; GLS—global longitudinal strain; SV—stroke volume; E—peak early-diastolic transmitral flow velocity; A—peak late-diastolic transmitral flow velocity; LAD—left atrial diameter (antero-posterior); AoR—aortic root dimension; RVD—right ventricular dimension (basal).

**Table 6:** Exercise-induced changes in echocardiographic parameters in the ERP+ and ERP− groups.

	ERP+	ERP−	*p*
**Left ventricular morphological parameters**			
Δ LVEDD, mm	0.3 ± 1.7	0.3 ± 2.2	0.998
Δ LVESD, mm	−4.6 ± 3.1	−4.5 ± 4.2	0.939
Δ IVST, mm	−0.2 ± 0.5	−0.5 ± 0.5	0.528
Δ PWT, mm	−0.1 ± 0.7	−0.2 ± 0.8	0.710
Δ RWT, %	−1.0 ± 3.7	−1.4 ± 3.7	0.751
Δ LVEDV, mL	−10.1 ± 11.8	−1.1 ± 13.2	0.020
Δ LVESV, mL	−6.6 ± 5.4	−3.0 ± 5.5	0.075
**Left ventricular systolic and diastolic parameters**			
Δ EF, %	2.6 ± 3.8	2.0 ± 3.8	0.635
Δ SF, %	10.5 ± 6.6	10.3 ± 8.2	0.931
Δ GLS	−1.7 ± 1.5	0.2 ± 1.5	0.652
Δ SV, mL	−4.8 ± 9.9	1.4 ± 8.9	0.031
Δ E, cm/s	18.5 ± 18.2	22.7 ± 17.0	0.452
Δ A, cm/s	16.2 ± 18.1	20.1 ± 25.6	0.594
Δ E/A	−0.1 ± 0.4	−0.1 ± 0.5	0.618
**Other morphological parameters**			
Δ LAD, mm	0.2 ± 3.3	−0.5 ± 3.4	0.483
Δ AoR, mm	−0.1 ± 0.7	0.0 ± 0.9	0.487
Δ RVD, mm	−0.4 ± 2.6	0.1 ± 1.8	0.534

Data are presented as means ± standard deviation. The Δ symbol represents the change in echocardiographic parameters calculated as the difference between values obtained immediately after exercise and those obtained at rest (Δ = post-exercise − rest). LVEDD—left ventricular end-diastolic diameter; LVESD—left ventricular end-systolic diameter; IVST—interventricular septum thickness at end-diastole; PWT—left ventricular posterior wall thickness at end-diastole; RWT—relative wall thickness; LVEDV—left ventricular end-diastolic volume; LVESV—left ventricular end-systolic volume; EF—ejection fraction; SF—shortening fraction; GLS—global longitudinal strain; SV—stroke volume; E—peak early-diastolic transmitral flow velocity; A—peak late-diastolic transmitral flow velocity; LAD—left atrial diameter (antero-posterior); AoR—aortic root dimension; RVD—right ventricular dimension (basal).

## Data Availability

The dataset used for the current study is available from the corresponding authors on reasonable request.
